# Outcome of Sleeve Gastrectomy Versus Single Anastomosis Sleeve Ileal Bypass on the Cardiac Functions and Rhythm Disturbance

**DOI:** 10.1007/s11695-025-07928-z

**Published:** 2025-06-26

**Authors:** Emad M. Abdelrahman, Mohamed Said Ghali, Amr G. Mohamed, Fatma Elhady, Shaimaa A. Habib, Asmaa Ahmed Ali, Zeyad Mohsen Elbagoury, Ahmed A. Dawood, Mahmoud A. Negm, Mahmoud Rizk, Osama R. Abdelraouf, Ahmed A. Elshora

**Affiliations:** 1 General Surgery Department, Faculty of Medicine, Benha University, Benha, Egypt; 2 General Surgery Department, Faculty of Medicine, Ain-Shams University, Cairo, Egypt; 3 Department of Surgery, Hamad Medical Corporation, Qatar, Qatar; 4 Cardiology Department Faculty of Medicine for Girls, Al-Azhar University, Cairo, Cairo, Egypt; 5 Department of Anesthesia and Surgical ICU, Faculty of Medicine, Benha University, Benha, Egypt; 6 Department of Internal Medicine, Faculty of Medicine, Benha University, Benha, Egypt; 7 Physical Therapy Program, Batterjee Medical College, Jeddah, 21442 Saudi Arabia; 8 General Surgery Department, Faculty of Medicine, Tanta University, Tanta, Egypt

**Keywords:** LSG, SASI, Cardiac functions, Rhythm disturbance

## Abstract

**Background:**

Cardiovascular hemodynamics, electrophysiological characteristics, and heart anatomy are all negatively impacted by obesity. The aim of this study is to compare the impact of sleeve gastrectomy versus single anastomosis sleeve ileal bypass on cardiac functions and rhythm disturbance.

**Methods:**

The current study included 78 patients who were allocated into two equal groups. Group A (*n* = 39) underwent laparoscopic sleeve gastrectomy (LSG), while group B (*n* = 39) underwent single anastomosis sleeve ileal bypass (SASI). Follow-up was designed for 6 and 12 months for cardiac functions and rhythm disturbance.

**Results:**

The patients’ mean age in the current study was 41.6 ± 6.88 and 43.2 ± 7.54 in groups A and B, respectively. There was a statistically significant longer operative time in patients who underwent SASI in comparison with those who underwent LSG (*P* < 0.001*). The %EWL was significantly higher in the SASI group at 6 and 12 months follow-up (*P* < 0.001*) QTC m sec and QT dispersion were significantly decreased within and between both groups after 6 and 12 months. There was a statistically significant improvement in the rhythm disturbance in both groups, mainly in group B, reported as a decrease in the overall AF with its subtypes in both groups. There was a statistically significant increase in the E/A ratio in both groups after 6 and 12 months follow-up, with no significant difference between both groups. There was an increase in LVEF in both groups, but it did not reach a significant value.

**Conclusions:**

LSG and SASI seem to be effective techniques in improving cardiac functions and overall AF in obese patients.

## Introduction

Obesity is a prevalent disease all over the world. The prevalence of obesity has almost doubled in the past 30 years [1]. Numerous co-morbidities, including obstructive sleep apnea syndrome (OSAS), hypertension, and cardiovascular disease (CVD), are linked to obesity [2].

Higher risks of atrial and ventricular arrhythmias, as well as sudden cardiac mortality, are associated with obesity. Obesity frequently co-occurs with diabetes, hypertension, and sleep apnea, all of which are separate risk factors for cardiac arrhythmias. However, there is strong evidence that weight loss can be a prophylactic measure to lower the occurrence of arrhythmias [3].

Cardiovascular hemodynamics, electrophysiological characteristics, and heart anatomy are all negatively impacted by obesity [4, 5]. QTc dispersion, P-wave lengthening, and QTc interval prolongation are all linked to obesity, as are rhythm abnormalities including atrial fibrillation. Consequently, cardiac arrhythmias and even sudden cardiac death are more likely to occur in obese people [6].

For patients with extreme obesity, bariatric surgery has been identified as the most successful and long-lasting long-term weight loss strategy [7]. Laparoscopic sleeve gastrectomy (LSG) is the first gold standard bariatric operation, performed most frequently these days, with a quick recovery and a shorter hospital stay [8–10].

In a new technique created by Brazilian surgeon Santoro and associates, a side-to-side gastro-ileal anastomosis is carried out following a sleeve gastrectomy [11]. However, the International Federation for the Surgery of Obesity and Metabolic Disorders and ASMBS (American Society for Metabolic and Bariatric Surgery) have not yet authorized this procedure [12]. Mahdy et al. subsequently dubbed this surgery the single anastomosis sleeve ileal (SASI) bypass [13]. Several studies have shown that bariatric surgery improves the anatomy and function of the heart [14].

The grey area about the effect of SASI in controlling the cardiac arrhythmia and its impact on the cardiac functions has motivated the authors to conduct this study in comparison with the standard LSG.

## Patients and Methods

### Study Design

Following the ethical perspectives of the Helsinki Declaration, the current study, a randomized prospective clinical study, was conducted after obtaining informed consent from all included patients. The present study included 78 obese patients with cardiac dysfunctions and rhythm disturbances.

Exclusion criteria included patients with documented ischemic heart disease, significant valve disease, severe chronic obstructive pulmonary disease, or pulmonary hypertension. Patients with ASA scores greater than 3 were excluded from the study. Patients who underwent previous bariatric procedures or patients who refused to be included in the study were also excluded.

The current study was conducted at the General Surgery Departments in Benha, Ain-Shams, Tanta University Hospitals, and the Cardiology Department et al.-Azhar University—throughout the period from January 2021 to March 2024. Eligible patients were randomly allocated into one of two equal groups. Group A (*n* = 39) underwent laparoscopic sleeve gastrectomy, while group B (*n* = 39) underwent SASI procedure. Follow-up was designed for 12 months postoperative for weight loss and control of preoperative heart rhythm disturbance and cardiac functions.

Randomization was done using specific software (Random Allocation Software 1.0, 2011). This randomization was conducted by an independent investigator.

### Preoperative Evaluation

A routine comprehensive history was taken with great concern about the cardiac symptoms. All patients had clinical examinations, particularly for blood pressure. Weight, height, and BMI were then calculated. Then, patients were also evaluated preoperatively for cardiac functions and arrhythmia via the following:

- Electrocardiogram (ECG) for detection of arrhythmia, P-wave abnormality, QT prolongation, QT dispersion.
- 24-h ambulatory ECG (Holter) monitoring for the detection of different types of arrhythmias.
- Conventional transthoracic echo-Doppler study before surgery and 6 and 12 months later. All parameters were acquired in accordance with the American Society of Echocardiography guidelines. Measurements were performed regarding interventricular and posterior wall thickness, left ventricular volumes, ejection fraction (2D EF), and E/A ratio for the flow through the mitral valve.
- 2D speckle tracking echocardiography: the assessment of LV global longitudinal strain (LV-GLS) was conducted using 2D speckle tracking echocardiography. The cutoff values that we employed were 20.6% for LV-GLS.

### Preoperative Preparation

Before the operation, each patient received anticoagulation and antibiotic prophylaxis.

All patients received premedication with IV midazolam (0.05 mg/kg) and fentanyl (2 mic/kg). General anesthesia was applied with IV propofol (2 mg/kg), atracurium (0.5 mg/kg), and maintenance with inhalational sevoflurane at 2 MAC until the end of the operation. Any arrhythmias that occur intraoperatively can be managed by deepening anesthesia, giving analgesia, and if there is no response, IV lidocaine. 0.5 mg/kg can be given. After recovery, the patient was transferred to the postoperative ICU.

### Surgical Procedure

#### Group A LSG (Fig. 1)

**Fig. 1:**
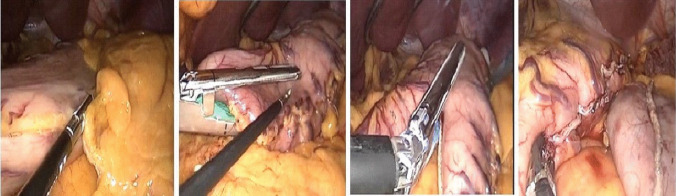
LSG

The standard 5-port technique was used. A 5-mm epigastric trocar (as a liver retractor), 10-mm supraumbilical (for the camera), a 15-mm left hypochondrial, a 12-mm right hypochondrial (two working ports), and a 5-mm left anterior axillary line subcostal port (assistant). Using a harmonic scalpel, the larger curvature was first de-vascularized 5 cm from the pylorus until the fundus was fully mobilized 2 cm from the angle of His. Using a Covidien linear stapler, a single green reload of 60–4.8 mm was used to staple, followed by blue reloads of 60–3.5 mm. Groups A and B underwent this procedure, while group B underwent omentopexy using a 2–0 PDS along the stable line. A leak test using methylene blue was done intraoperatively. Closure over the splenic drain was completed. The patients started oral sips 6–8 h postoperatively.

#### Group B (SASI) (Fig. 2)

**Fig. 2:**
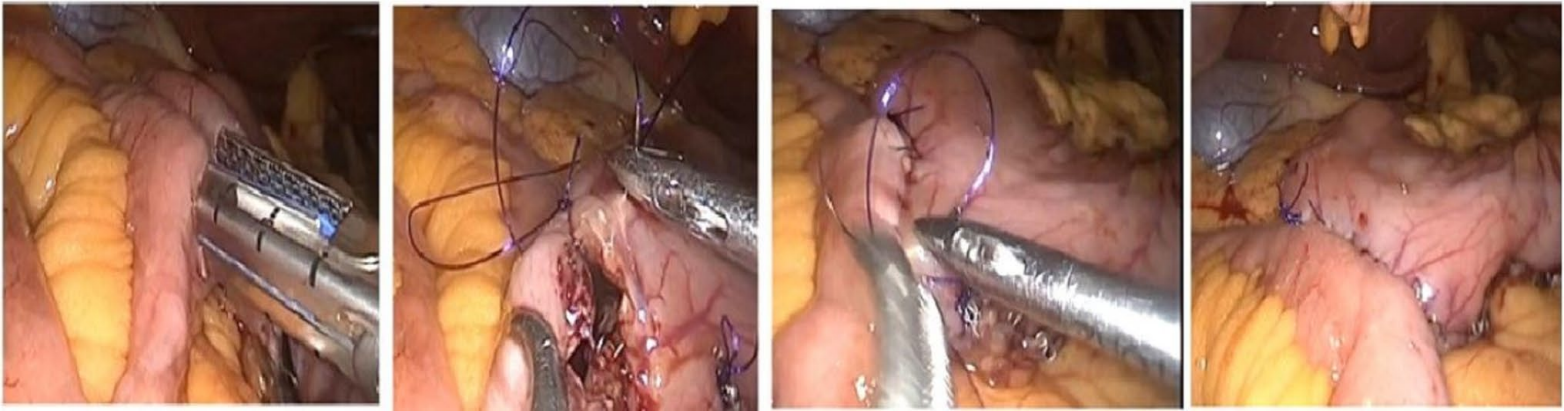
SASI

A 10-mm umbilical Visiport was used to produce pneumoperitoneum. To insert the liver retractor, a 5-mm trocar was inserted under the xiphoid process. For the surgeon's instruments, 12- and 15-mm trocars were inserted on the right and left middle clavicular lines, respectively. On the left anterior auxiliary line, a second 5-mm trocar was inserted for support. In order to decompress the stomach, an oral Ryle’s tube was inserted. Dissection then began on the larger curve, 5 cm from the pylorus up to the cardioesophageal junction, and continued until the gastric fundus was fully mobilized. The stomach was then resected using linear staplers. Methylene blue was used to examine the hemostasis and staple line. The patient was placed in the Trendelenburg position following the construction of the sleeved gastric tube. The patient’s transverse mesocolon was pulled back towards his or her head, and the small intestine’s distance from the ileocecal junction was measured at 250 cm (Fig. 2b). Then, using a 45-mm linear stapler, an antecolic side-to-side gastrojejunostomy of 3–3.5 cm was performed at the posterior wall of the region between the antrum and the body of the stomach. An uninterrupted Vicryl 2/0 stitch was used to seal the gastroenterostomy. The leak test was carried out by injecting 50–100 ml of methylene blue into the gastric pouch. Drains were left in place for 24 h.

Starting 4 weeks after surgery, all of our patients must follow the following regimen: ferrous fumarate (210 mg) once daily; Adcal D3® chewable tablets twice daily; 400 units of vitamin D and B12 injections every 12 weeks; and one daily multivitamin tablet.

### Postoperative Care and Follow-up

#### For Both Groups

Patients were advised to stroll around early in the few hours after surgery. Oral fluids were allowed 4 to 6 h after full recovery. The duration of each patient's hospital stay was recorded. During the first 24 h following surgery, patients were given omeprazole, 1 g of cefotaxime, and intravenous fluids. On-demand intravenous pethidine or paracetamol were used to treat the pain. On the third postoperative day, the patients were sent home. Proton pump inhibitors for 3 months, a liquid and soft diet for 2 weeks, and daily oral supplements of vitamin B12, calcium carbonate, vitamin D3, and iron were prescribed. The dosage of these supplements might need to be changed throughout the follow-up.

To monitor the surgery results, patients were requested to come in for evaluation every 2 weeks for the first 3 months and then every 3 months for the following year.

For the cardiac functions and rhythm, follow-up was planned after 6 and 12 months using ECG, ECHO, and Holter.

### Outcome and Follow-Up

#### Primary Outcome

The primary outcome was a successful LSG and SASI procedure with minimal postoperative complications.

#### Secondary Outcome

The secondary outcome was the potential remission or improvement of cardiac functions and heart rhythm.

### Statistical Analysis

The sample size required to achieve a power of 1 − *β* = 0.80 (80%) for the Spearman’s correlation at level *α* = 0.05 (5%), under these assumptions, amounts to 39 in each group (G*power, version 3.1).

For statistical analysis, SPSS, version 25 (IBM Corp., Armonk, NY, USA), was utilized. For quantitative factors defined by mean and SD, the Student’s *t*-test was employed. For qualitative data that were expressed as frequency with percentage, the χ2 test was employed. *P*-values below 0.05 were regarded as significant.

## Results

The patients’ mean age in the current study was 41.6 ± 6.88 and 43.2 ± 7.54 in groups A and B, respectively. AF was the most common type of arrhythmia, presented in 69.23% in group A and 71.79% in group B, followed by premature atrial contractions. There were no reported significant differences between both groups regarding the incidence of different types of arrhythmias. 79.48% and 84.61% in groups A and B, respectively, used antiarrhythmic drugs, while anticoagulants were used in 61.53% and 66.66% in groups A and B, respectively. Other sociodemographic data and comorbidities are reported in Table 1.

**Table 1:** Demographic data, baseline cardiac arrhythmia types, and medications used

Variables		Group A LSG *n* = 39	Group B SASI *n* = 39	*P* value
Age (years)	Mean ± SD	41.6 ± 6.88	43.2 ± 7.54	0.16
Gender Females Males	*n* (%) *n* (%)	26 (66.66%) 13 (33.33%)	28 (71.79%) 11 (28.2%)	0.28
BMI (kg/m^2^)	Mean ± SD	45.92 ± 4.98	47.78 ± 4.56	0.09
Comorbidities				
HTN	*n* (%)	8 (29.62%)	9 (23.1%)	0.17
DM	*n* (%)	11 (28.2%)	13 (33.33%)	0.32
Dyslipidemia	*n* (%)	13 (33.33%)	15 (38.46%)	0.63
Arrhythmia type				
AF total number Subtypes 1. Paroxysmal AF 2. Persistent AF 3. Long standing persistent AF	*n* (%) *n* (%) *n* (%) *n* (%)	27 (69.23%) 8 (29.62%) 17 (62.9)% 2 (7.4)%	28 (71.79%) 7 (25%) 19 (67.85)% 2 (7.1%)	0.76 0.19 0.31 0.92
Premature ventricular contractions	*n* (%)	2 (5.13)%	1 (2.56%)	0.06
Premature atrial contraction	*n* (%)	9 (23.1%)	8 (20.5%)	0.16
Supraventricular tachycardia	*n* (%)	1 (2.56%)	2 (5.13)%	0.06
Medications				
Antiarrhythmic medications	*n* (%)	31 (79.48)	33 (84.61)	0.93
Anticoagulants	*n* (%)	24 (61.53)	26 (66.66)	0.085

Table 2 shows a statistically significant longer operative time in patients who underwent SASI in comparison with those who underwent LSG (*P* < 0.001*). No intraoperative complications were reported in either group. Paralytic ileus occurred significantly in SASI (*P* < 0.001*), while no statistically significant difference was reported with regard to stable line bleeding and postoperative pneumonia. No statistically significant difference regarding hospital stay between both groups was reported.

**Table 2:** Operative data and postoperative complications

Variables		Group A LSG *n* = 39	Group B SASI *n* = 39	*P* value
Operative time (min)	Mean ± SD	45.5 ± 6.5	78.24 ± 11.22	< 0.001*
Intraoperative complications	***n* (%)**	0 (0.0%)	0 (0.0%)	**1.00**
Postoperative complications				
Staple line bleeding	***n* (%)**	1 (2.56%)	1 (2.56%)	**1.00**
Paralytic ileus	***n* (%)**	0 (0.0%)	2 (5.13)%	< 0.001*
Pneumonia	***n* (%)**	1 (2.56%)	2 (5.13)%	**0.06**
ICU admission	***n* (%)**	1 (2.56%)	2 (5.13)%	**0.06**
Post-operative mortality	***n* (%)**	0 (0.0%)	0 (0.0%)	**1.00**
Hospital stay (day)	Mean ± SD	2.1 ± 0.6	2.43 ± 0.7	**0.073**

Table 3 reports a statistically significant decrease in the BMI in both groups at 6 and 12 months follow-up when compared to the baseline, with more reported weight loss in patients who underwent SASI operation. The %EWL, as well as The TWL%, was significantly higher in group B at 6 and 12 months follow-up (*P* < 0.001*).

**Table 3:** Follow-up of BMI baseline and %EWL: ECG changes at 6 months and 12 months within and in between both groups

Variable	Group	Baseline	6 months	12 months	Baseline vs. 6 month	Baseline vs. 12 month	6 month vs. 12 month
BMI (kg/m^2^) Mean ± SD	**LSG group**	45.92 ± 4.98	**36.2 ± 5.22**	**29.23 ± 4.12**	< 0.001^*^	< 0.001^*^	< 0.001^*^
	**SASI group**	47.78 ± 4.56	32.78 ± 1.89	25.96 ± 2.42	< 0.001^*^	< 0.001^*^	< 0.001^*^
	***P* value**	0.09	0.012*	0.024*			
% TWL	**LSG group**		**21.7 ± 2.2%**	**36.5 ± 3.1**			< 0.001^*^
	**SASI group**		30.8 ± 3.24%	44.8 ± 2.7%			< 0.001^*^
	***P* value**		< 0.001*	< 0.001*			
%EWL Mean ± SD	**LSG group**		47.6 ± 4.4%	80.9 ± 5.1%			< 0.001^*^
	**SASI group**		65.8 ± 5.2	95.6 ± 4.23			< 0.001^*^
	***P* value**		< 0.001^*^	< 0.001^*^			
ECG changes							
P-wave abnormalities *n* (%)	**LSG Group**	11(28.20%)	7 (17.95%)	5 (12.8%)	0.01^*^	< 0.001^*^	0.01^*^
	**SASI Group**	10(25.64%)	5 (12.8%)	2 (5.13%)	< 0.001^*^	< 0.001^*^	< 0.001^*^
	***P* value**	0.28	0.01*	0.01*			
QTC m sec (mean ± SD)	**LSG group**	496 ± 19.6	438 ± 8.5	422 ± 7.8	< 0.001^*^	< 0.001^*^	0.12
	**SASI group**	511 ± 15.9	432 ± 9.4	418 ± 8.9	< 0.001^*^	< 0.001^*^	0.072
	***P* value**	0.27	0.19	0.082			
QT dispersion > 40, *n* (%)	**LSG group**	28(71.7%)	18 (46.15%	14 (35.9%)	< 0.001^*^	< 0.001^*^	0.01^*^
	**SASI group**	27(69.2%)	13 (33..3%)	8 (20.5%)	< 0.001^*^	< 0.001^*^	< 0.001^*^
	***P* value**	0.31	< 0.001^*^	< 0.001^*^			

ECG changes were reported in Table 3, where there was a significant decrease in the P-wave abnormalities in both groups, with more improvement in group B at 6 and 12 months when compared to the baseline. QTC and QT dispersion were significantly decreased within and between both groups after 6 and 12 months.

Table 4 reports a statistically significant decrease in the number of cases suffering from overall AF and the paroxysmal and persistent subtypes after 6 and 12 months follow-up (*P* ≤ 0.001^*^) with a significant decrease in group B when compared with group A. A subsequent increase in the number of patients de-escalated AAT and anticoagulants.

**Table 4:** Follow-up types of arrhythmia by Holter recording 24 h at 6 and 12 months within and in between both groups

Variable	Group	Baseline	6 months	12 months	Baseline vs. 6 month	Baseline vs. 12 month	6 month vs. 12 month
Arrhythmia outcomes							
Overall AF	**LSG group**	27 (69.23%)	19 (48.71%)	17 (43.58%)	< 0.001^*^	< 0.001^*^	**0.08**
	**SASI group**	28 (71.79%	16 (41.02%)	11 (28.2%)	< 0.001^*^	< 0.001^*^	< 0.001^*^
	***P* value**	0.76	0.046*	< 0.001^*^			
Paroxysmal AF	**LSG group**	8 (29.62%)	6 (22.2%)	5 (18.52%)	0.047*	0.014*	0.07
	**SASI group**	7 (25%)	4 (14.3%)	2 (7.14%)	0.021^*^	0.001^*^	0.017^*^
	***P* value**	0.19	0.041*	< 0.001^*^			
Persistent AF	**LSG group**	17 (62.9) %	12 (44.4%)	11 (40.7%)	< 0.001^*^	< 0.001^*^	0.19
	**SASI group**	19 (67.85)%	11 (39.3%)	8 (28.6%)	< 0.001^*^	< 0.001^*^	0.031*
	***P* value**	0.31	0.24	0.01*			
Long standing persistent AF	**LSG group**	2 (7.4)%	1 (3.7%)	1 (3.7%)	0.06	0.06	1.00
	**SASI group**	2 (7.1%)	1 (3.55%)	1 (3.55%)	0.06	0.06	1.00
	***P* value**	0.92	1.00	1.00			
Premature ventricular contractions	**LSG group**	2 (5.13)%	2 (5.13)%	2 (5.13)%	1.00	1.00	1.00
	**SASI group**	1 (2.56%)	1 (2.56%)	1 (2.56%)	1.00	1.00	1.00
	***P* value**	0.06	0.06	0.06			
Premature atrial contraction	**LSG group**	9 (23.1%)	7 (17.94%)	7 (17.94%)	0.43*	0.43*	**1.00**
	**SASI group**	8 (20.5%)	5 (12.82%)	2 (5.13)%	< 0.001^*^	< 0.001^*^	< 0.001^*^
	***P* value**	0.16	0.01*	< 0.001^*^			
Supraventricular tachycardia	**LSG group**	1 (2.56%)	1 (2.56%)	1 (2.56%)	1.00	1.00	1.00
	**SASI group**	2 (5.13)%	2 (5.13)%	2 (5.13)%	1.00	1.00	1.00
	***P* value**	0.06	0.06	0.06			
De-escalation of AAT	**LSG group**		17 (54.8%)	22 (71%)			0.01^*^
	**SASI group**		22 (66.7%)	27 (81.8%)			0.01^*^
	***P* value**		0.01^*^	0.01^*^			
De-escalation of anticoagulant	**LSG group**		9 (37.5%)	12 (50%)			0.01^*^
	**SASI group**		14 (53.8%)	19 (73.1%)			< 0.001^*^
	***P* value**		< 0.001^*^	< 0.001^*^			

Table 5 shows a significant improvement in posterior wall thickness, interventricular septal thickness, and relative wall thickness, with improvement in end-systolic and end-diastolic volume in both groups at 6 and 12 months when compared to the initial reports (< 0.001*).

**Table 5:** Follow-up types of cardiac functions by ECHO at 6 and 12 months within and in between both groups

Variable	Group	Baseline	6 months	12 months	Baseline vs. 6 month	Baseline vs. 12 month	6 month vs 12 month
Septal wall thickness, mm *N* = 6–9 mm female N6-10 mm male	**Group A**	11.45 ± 0.95	9.3 ± 0.8	8.95 ± 1.1	< 0.01^*^	< 0.01^*^	0.083
	**Group B**	11.56 ± 1.1	9.1 ± 0.5	9.0 ± 0.88	< 0.01^*^	< 0.01^*^	0.076
	***P* value**	0.78	0.29	0.11			
Posterior wall thickness, mm *N* = 6–9 mm Female 6–10 mm Male	**Group A**	10.95 ± 1.1	9.1 ± 0.91	9.0 ± 0.76	0.034*	0.041*	0.45
	**Group B**	10.69 ± 1.3	8.85 ± 0.72	8.76 ± 0.69	0.023*	0.038*	0.38
	***P* value**	0.92	0.79	0.66			
LV end-diastolic volume, mL *N* = 106 ± 22	**Group A**	126 ± 33	111 ± 24	110 ± 23	< 0.01^*^	< 0.01^*^	0.42
	**Group B**	128 ± 32	109 ± 22	108 ± 19	< 0.01^*^	< 0.01^*^	0.23
	***P* value**	0.12	0.17	0.24			
LV end systolic volume ml *N* = 41 ± 10	**Group A**	43 ± 16	34 ± 11	32 ± 9	< 0.01^*^	< 0.01^*^	0.09
	**Group B**	42 ± 15	35 ± 11	33 ± 10	< 0.01^*^	< 0.01^*^	0.13
	***P* value**	0.24	0.27	0.19			
Ejection fraction (EF%)2D *N* = 62 ± 5	**Group A**	59 ± 5	62 ± 7	62.5 ± 4	0.32	0.21	0.089
	**Group B**	57 ± 3	61 ± 4	62 ± 3.7	0.41	0.14	0.12
	***P* value**	0.13	0.18	0.086			
E/A ratio	**Group A**	0.84 ± 0.1	1.09 ± 0.17	1.1 ± 0.22	< 0.01^*^	< 0.01^*^	0.15
	**Group B**	0.85 ± 0.09	1.12 ± 0.29	1.15 ± 0.24	< 0.01^*^	< 0.01^*^	0.19
	***P* value**	0.12	0.09	0.082			
LV S GLS (%) −18 ± 2.5	**Group A**	− 14.2 ± − 2.1	− 18.2 ± − 2.4	− 18.5 ± − 2.2	< 0.01^*^	< 0.01^*^	0.14
	**Group B**	− 14.3 ± − 2.34	− 18.1 ± − 2.1	− 18.9 ± − 2.3	< 0.01^*^	< 0.01^*^	0.19
	***P* value**	0.06	0.085	0.78			

There was a statistically significant increase in the E/A ratio in both groups after 6 and 12 months of follow-up up with no significant difference between the groups. There was an increase in LVEF in both groups, but it did not reach a significant value (Table 5).

## Discussion

Bariatric surgery offers a widely accepted alternative technique for managing obesity compared to lifestyle therapies and medicine, with a longer-lasting initial weight loss [12].

In the current study, there was a statistically significant lesser operative time in LSG than in SASI, and this can be explained by the additional surgical steps in SASI.

The reported time for SASI was less than that of other studies [13, 15]; this is assumed to be due to the different learning curve and available energy resources.

The average length of stay at the hospital was 2.43 ± 0.7 for the SASI group, with no significant difference when compared to the LSG group, matching earlier research [15, 16]. Ileus was evident in the SASI group when compared to the LSG group; otherwise, no significant difference in the postoperative complications was reported.

In the current study, there was a significant decrease in BMI within and in between both groups after 6 and 12 months follow-up. This matches the results of Emile et al. [17] who reported EWL% in patients who underwent SASI after 1 year as more than 83%. Other authors [18–21] reported EWL% of more than 90%. The current results, although, were higher than the results of Madyan et al. [22], who reported EWL% of 65.2% at 12 months follow-up. Many variables play an important role in weight loss, including the preoperative BMI, the common channel length, and the size of the gastro-ileal anastomosis in the SASI group, as anastomoses less than 2 cm are more likely to develop stenosis and occlusion. The gastro-ileal anastomosis should be 3–4 cm in size.

Numerous studies have shown that bariatric surgery improves the anatomy and function of the heart [7]. The effect of obesity on cardiac electrophysiology is quite complex, including delays in atrial and ventricular repolarization. The repolarization defects in obese people are mostly attributed to insulin resistance [22, 23].

Obesity is also associated with the lengthening of the P-wave and rhythm disorders like AF [6]. QTc prolongation dispersion is usually presented in hypertensive and obese patients with left ventricular (LV) hypertrophy, [7] and this matches the initial reports of the included patients in the current study. There is an association between Insulin resistance, hyperinsulinemia, and obesity predisposing to QT prolongation [23]. This can occur due to three main factors. The first is evident in hyperpolarizing the plasma membranes of both excitable and non-excitable tissues, which leads to the prolongation of the QT interval that occurs with hyperinsulinemia. Secondly, hyperinsulinemia causes hypokalemia, which is a known factor that can induce the prolongation of the QTc interval. Thirdly, hypertension and left ventricular hypertrophy are associated with delayed ventricular repolarization [6, 24].

In the current study, there was a statistically significant improvement in the ECG changes associated with obesity in both groups, including a decrease in the P-wave abnormalities, with more improvement in group B at 6 and 12 months. QTC m sec and QT dispersion were significantly decreased within and between both groups after 6 and 12 months, and this matched the results of Sanches E et al*.* [6] and other studies [25, 26] who reported a decrease in the QT interval, QT interval dispersion, or P-wave dispersion after bariatric surgery, irrespective of follow-up length or its type, malabsorptive or restrictive, with significant improvement in malabsorptive procedures matching the current results, where a statistically significant improvement in the QTc was reported in patients who underwent SASI.

Guiraud et al*.* [27] reported that individuals participating in a cardiac rehabilitation exercise-training program with BMI loss had a significant decrease in QT dispersion. Corbi et al*.* [28] reported that a low-calorie diet and voluntary activity program with a mean reduction in BMI of 5 kg/m^2^ resulted in dramatically decreased QT dispersion. Omran et al*.* [29] reported that obese patients had a significantly longer QTc interval, and there is a substantial correlation between weight loss and improved QTc interval.

Excessive long or short QT interval is associated with irregular heart rhythms and cardiac death. Prolonged QTc raises the likelihood of ventricular arrhythmias, including deadly ventricular fibrillation, by causing premature action potentials during the late stages of depolarization [7].

P-wave dispersion is a sensitive and specific ECG predictor of AF. Ventricular repolarization improved significantly after weight loss ^**(5)**^. Atrial Fibrillation (AF) is the most prevalent arrhythmia. It affects 0.5% of people, and many of them are obese, predisposing to heart failure and stroke [30, 31]. Atrial arrhythmias, particularly paroxysmal atrial fibrillation, usually have increased P-wave duration and P-wave dispersion.

In the current study, there was a statistically significant improvement of the rhythm disturbance in both groups, mainly in Group B, reported as a decrease of the overall AF with its subtypes, with a subsequent increase in the number of patients that de-escalated AAT and anticoagulants. This matched the results of many studies [4, 5, 29] that presented the effect of bariatric surgery on cardiovascular diseases. This can be explained by remodeling the gastrointestinal tract through reduced stomach volume and reduced absorption of ingested nutrients due to shortening of the small bowel following bariatric surgery, with subsequent weight loss.

A decrease in weight or BMI can induce a decrease in EAT, which can be beneficial for the normalization of the QT interval [32].

In the current study, there was a statistically significant weight loss in both groups, with more weight loss in patients who underwent SASI, and all positive cardiac outcomes are linked to this significant weight loss. Obesity changes the anatomy and function of the heart, causing ventricular and septal hypertrophy, increasing the left ventricular mass (LVM) [7]. LV reverse remodeling usually follows bariatric surgery [33].

The current study demonstrated a significant improvement in posterior wall thickness, interventricular septal thickness, and relative wall thickness, with improvement in end-systolic and end-diastolic volume supporting increased filling and relaxation of the left ventricles in both groups, matching the results of Sargsyan et al*.* [7] who reported a significant improvement in left ventricular hypertrophy (LVH), reduced ventricular contractility, and coronary reserve, as well as a reduction in the inflammatory state that causes ventricular hypertrophy following bariatric surgery [4].

Diastolic dysfunction with compromised myocardial relaxation is characterized by decreased early (E wave) and increased atrial LV filling (A wave). The current study demonstrated a statistically significant increase in the E/A ratio in both groups after 6 and 12 months, matching the results of many authors [34] who have reported similar outcomes following bariatric surgery.

Significant improvements in systolic function and a notable rise in LVEF are linked to rapid weight loss [35]. LVEF in both groups was increased in the current study, which is consistent with findings from several studies [7].

LVGLS was improved in both groups in the present study, which is used as a marker of improved left systolic mechanics and functions, matching the results of Sorimachi H et al*.* [36] who reported that postoperative increase in GLS is associated with a postoperative reduction in afterload and BMI.

## Conclusion

According to the current results, LSG and SASI seem to be effective techniques in improving cardiac functions and overall atrial fibrillation in obese patients.

### Limitations

Being to some extent novel, limited studies were carried out to detect the effect of SASI in controlling arrhythmias and improving cardiac functions in obese patients, and this represented an obstacle as there were limited studies to compare our results with them. Another point was the commitment of some patients to follow-up schedules and the strict follow-up parameters and assessment, especially the Holter.

## Data Availability

No datasets were generated or analysed during the current study.
